# Changes in Microvascular Morphology in Subcortical Vascular Dementia: A Study of Vessel Size Magnetic Resonance Imaging

**DOI:** 10.3389/fneur.2020.545450

**Published:** 2020-10-29

**Authors:** Hyeon-Il Choi, Chang-Woo Ryu, Songvin Kim, Hak Young Rhee, Geon-Ho Jahng

**Affiliations:** ^1^Department of Radiology, Neurology, Kyung Hee University Hospital at Gangdong, Seoul, South Korea; ^2^College of Medicine, Kyung Hee University, Seoul, South Korea; ^3^Department of Neurology, Kyung Hee University Hospital at Gangdong, Seoul, South Korea

**Keywords:** dementia, vascular - diagnosis, magnetic resonance imaging, cerebral small vessel diseases, microvessels, vessel size imaging

## Abstract

**Background:** Cerebral small vessel disease is the most common cause of subcortical vascular dementia (SVaD). Unfortunately, conventional imaging techniques do not always demonstrate the microvascular pathology that is associated with small vessel disease. The purpose of this study was to evaluate the changes in the microvascular structure of SVaD and to identify how the microvascular changes in vessel size, detected with imaging, affect the gray matter.

**Methods:** Ten SVaD patients and 12 healthy controls underwent vessel size imaging with gradient-echo and spin-echo sequences before and after contrast agent injection. Four microvessel index maps, including total blood volume fraction (BVf), mean vessel density (Q), mean vessel diameter (mVD), and vessel size index (VSI) were calculated. ROI value of each microvessel parameter was compared between SVaD patients and controls. Voxel-wise comparison of microvessel parameters was also performed to assess the regional difference. The relationship between the microvessel parameters in white matter and total gray matter volume (TGV) were assessed.

**Results:** Both mVD and VSI were significantly different between the SVaD and controls in the ROI-based comparisons (unpaired *t*-test, *p* < 0.05). mVD and VSI were significantly increased in the SVaD group at the subcortical, periventricular white matter, basal ganglia, and thalami compared with the controls (FDR corrected, *p* < 0.05). VSI in the white matter areas were significantly negatively correlated with TGV (*r* = −0.446, *p* < 0.05).

**Conclusions:** The increase of mVD and VSI in SVaD patients reflects the damage of the microvessels in the white matter, and these changes may lead to the damage of the gray matter.

## Introduction

Cerebrovascular disease is a major contributor to cognitive decline in the elderly even though it has proven to be somewhat elusive to diagnose. The most common cause of subcortical vascular dementia (SVaD) is cerebral small vessel disease (SVD), which typically manifests as white matter lesions and/or lacunes on brain imaging. SVD primarily distresses the small perforating arteries, being defined as vessels of <50 μm diameter supplying the deep brain structure. This SVD often occurs with dementia and worsens cognitive impairment. Therefore, dementia is becoming a big health burden (1).

SVD of the central nervous system begins with microangiopathies, such as arteriolosclerosis, lipohyalinosis, or fibrinoid necrosis. These pathologic changes lead to disturbance in the microcirculation and chronic hypoxia in the brain (2–4). The chronic hypoxia results in pathologic changes of white matter, including axonal loss, demyelination, and gliosis, which is represented as hyperintense signals on T2-weighted images (T2WI), which are called white matter hyperintensities (WMH) (2–4). Despite the importance of SVD, the dominant diagnostic tool for this diagnosis relies upon the visual inspection of conventional imaging and neuropsychological tests. Unfortunately, it remains difficult to discriminate the pathological changes underlying cognitive decline in SVD using conventional brain imaging, and brain damage is not always limited to the initially visible lesions (5).

Recently, multiple attempts have been made to characterize the microvasculature using perfusion and pharmacokinetics to measure the mean vessel caliber and microvessel density (*in vivo* on MRI). A concept of vessel size imaging originated from the difference in relative cerebral blood volume between the gradient-echo (GE) and spine-echo (SE) techniques in gliomas. The GE relaxation rate remains constant although the SE relaxation rate decreases with the vessel size. This vessel size imaging is obtained from the ratio of GE and SE relaxation rate changes (ΔR2^*^, ΔR2). These changes are induced by intravascular super-paramagnetic contrast agents (6).

So far, most studies applying this technique have been used to characterize cerebral tumor vascularization (6–9). This technique has also recently been shown to be useful when applied to ischemic stroke (10). Despite its potential in the assessment of microvascular pathology, to our knowledge, vessel size imaging has not been addressed in the diagnosis of SVD previously. This method provides non-invasive information regarding the morphological variations in microvascular structures. Therefore, this technique can provide useful information for SVD in SVaD. It might also have diagnostic benefit in SVaD.

The main goals of the present study were to investigate the change of the cerebral microvascular structures in SVaD patients compared to cognitively normal elderly using *in vivo* vessel size imaging and to identify how cerebral microvascular change in SVaD patients affects the gray matter. This effort may help to understand the pathophysiologic mechanism of the microvascular change in SVaD.

## Materials and Methods

### Subjects

This is a prospective, cross-sectional, case-control observation study. This study was approved by the institutional review board at our hospital. Informed consent was obtained. The SVaD patients were enrolled from the prospective registry of vascular cognition impairment of an institute between March 2017 and August 2018. Based on the NINDS-AIREN definition, the inclusion criteria were defined as follows: (1) age >60 years, (2) dementia: impairment of memory and of two or more cognitive domains, (3) focal neurologic signs, (4) large confluent WMH with/without lacunes on T2WI, and (5) Hachinski ischemic score >6 (11). All of the patients who presented with at least one of the following criteria were excluded: (1) any other cause of dementia based on the *Diagnostic and Statistical Manual of Mental Disorders-V* (DSM-V), such as Alzheimer disease (AD) or Lewy body disease; (2) systemic causes of subacute cognitive impairment; and (3) cortical and/or cortico-subcortical non-lacunar territorial infarcts and watershed infarcts, hemorrhages indicating large vessel disease, signs of normal pressure hydrocephalus, and specific causes of white matter lesions (e.g., multiple sclerosis, sarcoidosis, brain irradiation). Healthy controls were enrolled on a voluntary basis among subjects who attended a health-screening program. Control subjects were healthy volunteers without memory impairment or cognitive disorders who were recruited from the neurology clinics. They did not differ significantly in age, sex, or education from the patients with SVaD.

A total of 22 subjects including 10 SVaD patients (age 77.7 ± 7.0 years; median Korean-Mini Mental Status Examination [K-MMSE] 18.5, interquartile range [IQR]: 15–19) and 12 controls (age 74.3 ± 4.6 years; median K-MMSE 27, IQR: 26–28) were enrolled in this study. The participants also completed neuropsychological assessments, including the K-MMSE, global deterioration scale (GDS), and clinical dementia rating (CDR).

### MRI Acquisition

MRI data were acquired using a 3T scanner (Philips Healthcare, The Netherlands) in all subjects. Sagittal structural 3-D T1-weighted images (T1WI) were acquired using the magnetization-prepared rapid acquisition of gradient echo (MPRAGE) sequence with the following parameters: repetition time (TR) = 8.1 ms, echo time (TE) = 3.7 ms, flip angle (FA) = 8°, field of view (FOV) = 236 × 236 mm^2^, and voxel size = 1 × 1 × 1 mm^3^. In addition, the fluid-attenuated inversion recovery (FLAIR) image was acquired to examine any abnormality of the brain and to allow visual assessment of WMH. A diffusion-weighted imaging (DWI) sequence with *b* = 0 and 1,000 s/mm^2^ was also acquired to calculate the apparent diffusion coefficient (ADC) before contrast agent injection.

To calculate the R2 relaxation rates, a multiecho multislice (MEMS) turbo-spin-echo (TSE) sequence was scanned before and after contrast injection. The imaging parameters for the MEMS TSE imaging were as follows: TR = 11,072 ms, TEs = 16 + 32^*^n where *n* is the number of echoes, number of echoes = 7, FOV = 210 × 210 mm^2^, acquisition voxel size = 1.1 × 1.1 × 3 mm^3^, reconstruction voxel size = 0.4 × 0.4 × 3 mm^3^, reconstruction matrix size = 512 × 512, number of slices = 45, TSE factor = 14, and the scan time = 7 min 45 s. Furthermore, to calculate the R2^*^ relaxation rates, a MEMS gradient-echo (GRE) sequence was run before and after contrast injection. The imaging parameters for the MEMS GRE imaging were the same as those of the MEMS TSE sequence except the following: TR = 2,882 ms, TEs = 2 + 10^*^n where *n* is the number of echoes, number of echoes = 7, FA = 60°, and the scan time = 4 min 43 s. A dose of 0.13 mL Gadovist (Bayer Schering Pharma AG, Berlin, Germany) per kg body weight was injected at a speed of 5 mL/s with a time delay of 18 s.

### Mapping for Vessel Size Imaging Parameters

The R2 and R2^*^ relaxation rates were calculated by fitting the exponential decay curves from the MEMS TSE and MESE GE images, respectively. Δ*R*_2_ and $\Delta R_{2^{*}}$ were defined by the differences in the relaxation rate from before and after contrast injection. Based on ΔR2, Δ*R*2^*^, and ADC maps, the following four vessel size imaging parameters—total blood volume fraction (BVf), mean vessel density (Q), mean vessel diameter (mVD), and vessel size index (VSI)—were calculated according to the following equations:

$$\begin{array}{l} \;BVf=3/\left(4\pi \cdot \gamma \cdot \;x\;\Delta \cdot B0\right)\cdot \Delta R2^{*}\\ mVD=\Delta \;R2^{*}/\Delta R2\\ \;Q=\Delta R2^{*}/{\left(\Delta R2\right)}^{2/3}\\ \;VSI=0.424\cdot {\left(ADC/\gamma \cdot \Delta \;x\;\cdot B0\right)}^{1/2}\cdot {\left(\Delta R2^{*}/\Delta R2\right)}^{3/2}, \end{array}$$

where γ is the gyromagnetic ratio of hydrogen proton (Hz/T), B0 is the strength of the main magnetic field (i.e., 3T), and χ (in cgs units) is the change in intravascular magnetic susceptibility due to the administration of the paramagnetic contrast agent.

### Preprocessing of the Microvascular Index Maps

To compare the microvascular parameters between the controls and patients, the following preprocessing steps were performed using statistical parametric mapping software (SPM12, http://www.fil.ion.ucl.ac.uk/spm/). First, all parameter maps and FLAIR images of each subject were coregistered to 3-D T1WI. Next, the 3-D T1WI was spatially normalized into the brain template and segmented into gray and white matter using the cat12 tool (http://www.neuro.unijena.de/ca). All parameters and FLAIR images were then spatially normalized using the parameter of the deformation of 3-D T1WI. Finally, spatial smoothing was applied to all microvessel parameters and brain tissue volumes using the Gaussian kernel of an 8 × 8 × 8 mm^3^ full width at half maximum to voxel-based statistical analyses (Supplementary Figure 1).

### Statistical Analyses

Basic demographics and descriptive clinical data are presented as mean and standard deviation or median value and range. Differences between the SVaD and controls were evaluated using a two-tailed unpaired Student's *t*-test for continuous variables and the chi-square test or Mann-Whitney test for categorical variables. The *p* < 0.05 was considered statistically significant.

#### Region of Interest (ROI)-Based Analysis

**Figure 1:**
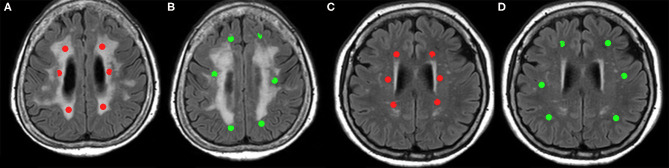
Six sphere ROIs 3 mm in diameter are manually assigned to subcortical vascular dementia (SVaD) patients and controls. **(A)** ROIs drawn in white matter hyperintensity (WMH) (red), and **(B)** ROIs drawn in normal-appearing white matter (NAWM) (green) in SVaD patients. **(C)** ROIs drawn in periventricular white matter (PVWM) (red), and **(D)** ROIs drawn in deep white matter (DWM) (green) in controls, which are matched with geometrically ROI drawn in SVaD patients.

To compare the microvessel parameters between the patients and controls, ROI analysis was performed. Six sphere ROIs with 3 mm in radius at the bilateral anterior, middle, and posterior periventricular white matter (PVWM) were manually placed in the WMH on FLAIR images using MRIcro (http://www.cabiatl.com/mricro/) software by a single operator. The other six ROIs were selected from normal-appearing white matter (NAWM) in deep white matter (DWM) in patients. To avoid the ROI including gray matter or different white matter, we constrained the maximum image intensity difference from the seed as 10. The ROIs were then superimposed in identical positions on six microvessel parameter maps to obtain microvessel parameter values. In the control group, six ROIs were also placed in bilateral anterior, middle, posterior PVWM, and six ROIs in DWM, which were geometrically matched WMH and NAWM of SVaD, respectively. Figure 1 shows how the ROIs were drawn in the white matter. The parameters of each ROI were obtained from the coregistered parameter maps.

We performed the following statistical analyses. First, values of ROI in SVaD patients and values of ROI in controls were compared by using an unpaired *t*-test. The *p* < 0.05 was considered statistically significant. Second, to determine the power of the microvessel parameters to discriminate between SVaD and controls, a stepwise discriminant analysis was performed using the ROI values as the independent variables. ROC curves and corresponding areas under the curve (AUC) were calculated to assess the sensitivity and specificity of the best discriminative variables (12).

#### Voxel-Based Group Analysis

Age and total intracranial volume (TIV) should be considered for voxel-based morphometric (VBM) analysis because these are regarded as independent variables that affect the gray matter volume. Several studies present that subjects with a larger brain are likely to exhibit increased gray matter volumes although not necessarily according to the same proportions (13, 14). Age is also a well-recognized factor affecting the measurement of gray matter volume in ROI and voxel-based methods (15, 16). Therefore, age and TIV were adjusted for voxel-based analysis in group comparison and regression and correlation statistics to scale the effect by age and TIV during the statistical analyses for gray matter volume. To compare the microvessel parameter maps between the patients and controls, a voxel-based, two-sample *t*-test was used with age and TIV as covariates. We applied a significance level of *p* = 0.05 with a false discovery rate (FDR) correction for multiple comparison clusters of at least 30 contiguous voxels for each measure. If there was a significant region that showed group difference in VBM analysis, the mean microvessel parameter values of these clusters were extracted from each subject to use as a variable for the next correlation analysis.

#### Correlation With Gray Matter Volumes

To explore the relationship between the microvascular characteristics and the gray matter volume, the values of the microvessel maps were extracted from the voxels that had more than 50% white matter volume. The voxel-based regression analysis was performed with the gray matter volume and the extracted microvessel parameter values. The threshold for statistical significance was set at an FDR-corrected *p* < 0.05.

Furthermore, the partial correlation coefficient was also calculated between microvessel parameters from white matter and TGV after adjusting for age and TIV. The *p* < 0.05 was considered statistically significant.

## Results

Basic information (age, sex, and TIV) of subjects did not show a difference between the two groups. Three neuropsychological tests (K-MMSE, GDS, and CDR) showed significant differences between the two groups (Table 1).

**Table 1:** Clinical characteristics of subjects.

	**SVaD (*n* =10)**	**Controls (*n* = 12)**
**Age**	77.7 ± 7.0	73.3 ± 4.6
**Sex**	male 4	male 6
**TIV (ml)**	1420.8 ± 102.4	1489.6 ± 143.2
K-MMSE^*^	17.6 ± 4.8	26.8 ± 2.0
GDS^*^	median 5 (range: 4–5)	median 3 (range: 2–3)
CDR^*^	median 2 (2)	median 0.5 (0–0.5)

*SVaD, subcortical vascular dementia; CN, control; K-MMSE, Korean-Mini Mental Status Examination; GDS, global deterioration scale; CDR, clinical dementia rating; TIV, total intracranial volume*.

* *means statistically significant difference (p < 0.05) between two groups*.

### ROI-Based Analysis

Because several SVaD patients did not have a large enough NAWM volume to be selected for drawing the ROI between the cortex and large confluent WMH, ROI subtraction for NAWM in patients' group were not conducted in 12 areas of 8 patients. Therefore, 56 ROIs in WMH and 48 ROIs in NAWM of patients' group, 72 ROIs in PVWM, and 72 ROIs in DWM of controls group were compared.

**Figure 2:**
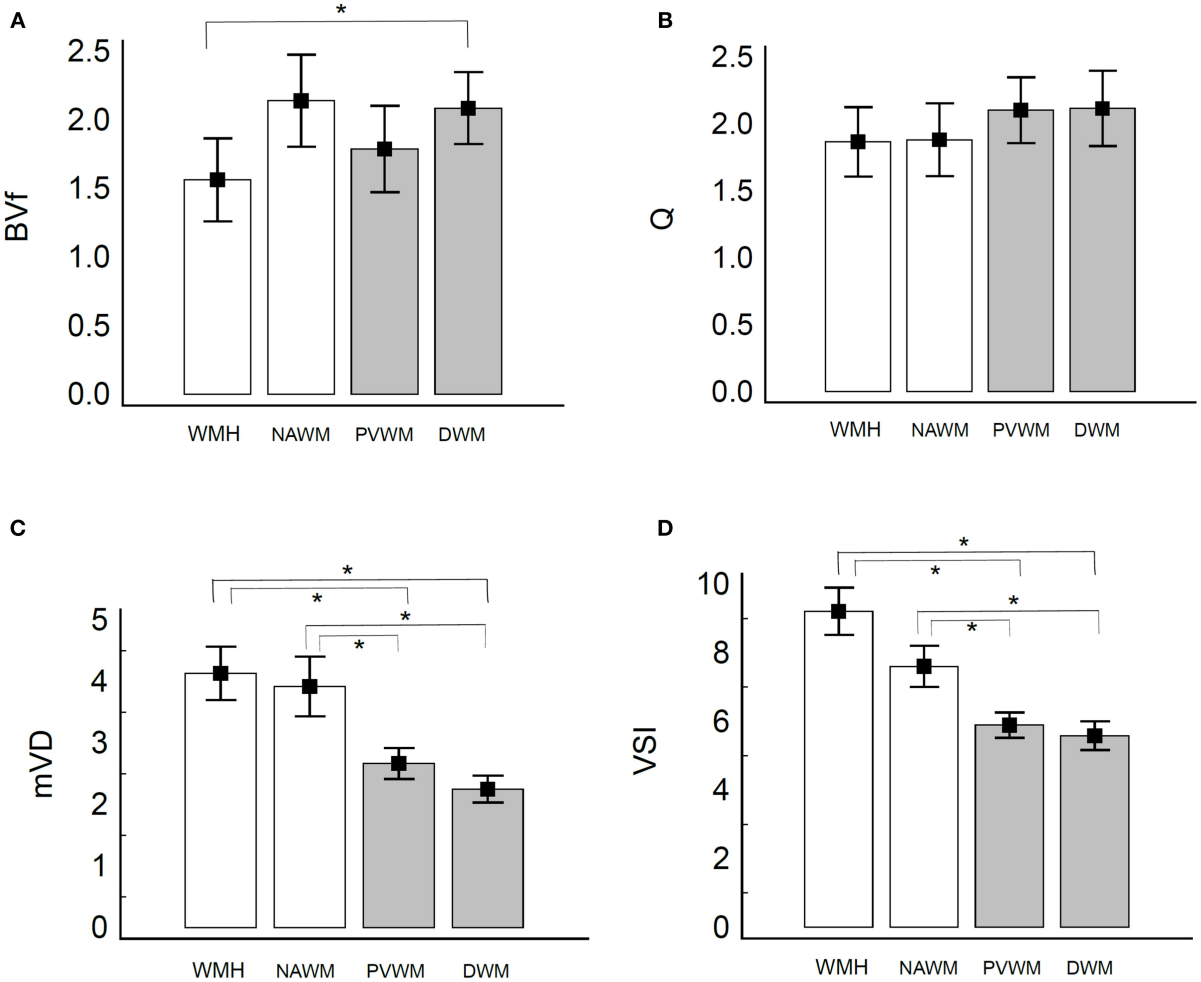
Bar graph of microvessel parameters of white matter in patients with SVaD and healthy controls. Vertical lines represent the standard deviation. **(A)** BVf, **(B)** Q, **(C)** mVD, and **(D)** VSI. The white bar presents value in patients with SVaD and the gray bar presents in the value in healthy controls. *indicate statistically significant differences (*P* < 0.05). The BVf is significantly lower in WMH of SVaD compared to DWM of the controls **(A)**. The Q value of white matter was not significantly different between the two groups **(B)**. The mVD and VSI of white matter are significantly higher in SVaD patients compared to controls regardless of the location **(C,D)**.

The ROI value of BVf was significantly lower in WMH of SVaD compared to DWM of the controls (1.562 ± 1.131 and 2.085 ± 1.116, respectively). However, the BVf in NAWM of SVaD (2.139 ± 1.154) was not significantly different compared to PVWM (1.787 ± 1.339) or DWM of controls (Figure 2A). In the comparison of Q value, WMH (1.865 ± 0.967) and NAWM (1.881 ± 0.935) of SVaD were not significantly different to PVWM (2.102 ± 1.045) or DWM (2.115 ± 1.195) in controls (Figure 2B). The ROI values of mVD in WMH (4.131 ± 1.621 μ*m*) and NAWM (3.917 ± 1.671 μ*m*) of the SVaD group were significantly higher than those in both PVWM (2.666 ± 1.078 μ*m*) and DWM (2.249 ± 0.936 μ*m*) of the control group (Figure 2C). The ROI values of VSI in WMH (9.211 ± 2.572 μ*m*) and NAWM (7.604 ± 2.060 μ*m*) in the SVaD group were also significantly higher than those in PVWM (5.891 ± 1.568 μ*m*) and DWM (5.584 ± 1.781 μ*m*) in the control group regardless of the location of the ROI (Figure 2D).

In the mono-exponential model, ROC analysis to discriminate between WMH of SVaD and PVWM of control demonstrated that mVD yielded an AUC of 0.792 and 0.864 in NAWM. In discrimination between WMH of SVaD and DWM of control, AUC of mVD and VSI were 0.861 and 0.877, respectively. AUC of mVD and VSI were 0.815 and 0.761 in the discrimination of NAWM of SVaD from DWM of controls. AUCs of mVD and VSI were under 0.75 (0.737 and 0.735, resepectively) in the discrimination of NAWM of SVaD from PVWM of controls. The AUCs of BVf and Q were <0.75 in the comparison between the patients and control groups (Table 2, Supplementary Figure 2).

**Table 2:** Results of ROC curve analyses for discriminating microvessel parameters in SVaD from those in controls.

	**WMH-SVaD vs. PVWM-control**		**WMH-SVaD vs. DWM-control**		**NAWM-SVaD vs. PVWM-control**		**NAWM-SVaD vs. DWM-control**	
**Parameter**	**AUC**	**95% CI**	**AUC**	**95% CI**	**AUC**	**95% CI**	**AUC**	**95% CI**
BVf	0.563	0.472–0.650	0.683	0.595–0.762	0.66	0.571–0.741	0.509	0.416–0.602
Q	0.562	0.472–0.650	0.556	0.466–0.644	0.628	0.539–0.712	0.549	0.539–0.712
mVD	0.792**^a^**	0.711–0.859	0.861**^c^**	0.789–0.916	0.737	0.648–0.813	0.815^e^	0.734–0.880
VSI	0.864**^b^**	0.793–0.918	0.877**^d^**	0.807–0.928	0.735	0.647–0.811	0.761^f^	0.674–0.834

*WMH, white matter hyperintensity; NAWM, normal appearing white matter; SVaD, subcortical vascular dementia; AUC, area under curve; SE, standard error; CI, confidence interval*.

a *Cutoff value, 2.863 μm; Sensitivity, 76.8; Specificity, 72.2*.

b *Cutoff value, 6.520 μm; Sensitivity, 82.1; Specificity, 76.4*.

c *Cutoff value, 2.377 μm; Sensitivity, 89.3; Specificity, 68.6*.

d *Cutoff value, 6.960 μm; Sensitivity, 78.6; Specificity, 83.3*.

e *Cutoff value, 2.477 μm; Sensitivity, 81.3; Specificity, 73.6*.

f *Cutoff value, 6.575 μm; Sensitivity, 68.8; Specificity, 76.4*.

### Voxel-Based Analysis

**Figure 3:**
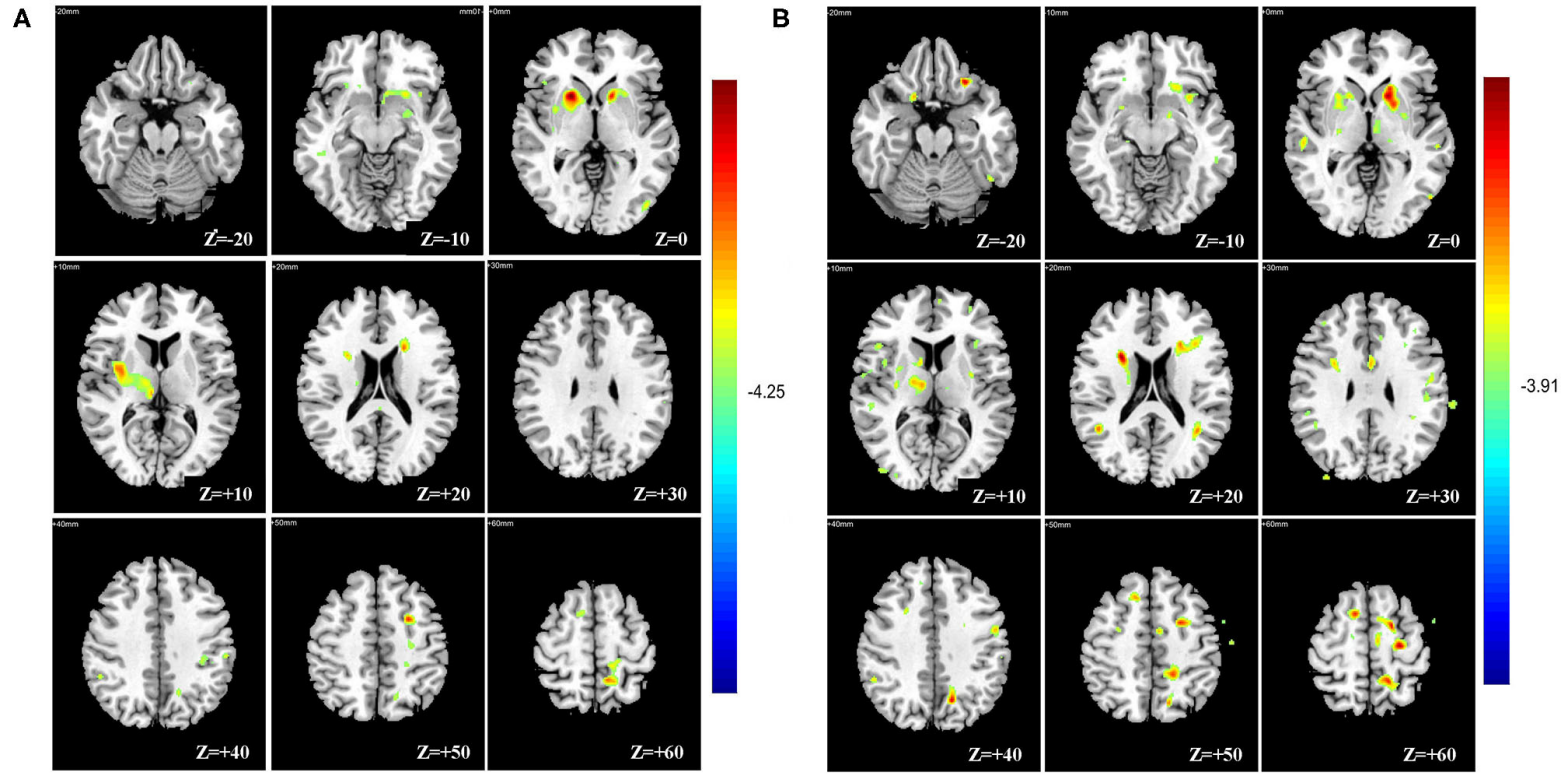
Voxel-based analysis showing multiple regional increases of mean vessel diameter (mVD) **(A)** and vessel size index (VSI) **(B)** in basal ganglia, thalami, periventricular, and juxtacortical white matter of the subcortical vascular dementia (SVaD) group compared with the control group.

The result of the two-sample *t*-test shows that mVD and VSI in the SVaD group were increased in the juxtacortical white matter, periventricular white matter, basal ganglia, and thalami compared to those of the control group (Figure 3). There were no areas to show any regional decrease in the mVD or VSI in the SVaD group. Q and BVf were not significantly different between the two groups.

### Correlation With Gray Matter Volume

**Figure 4:**
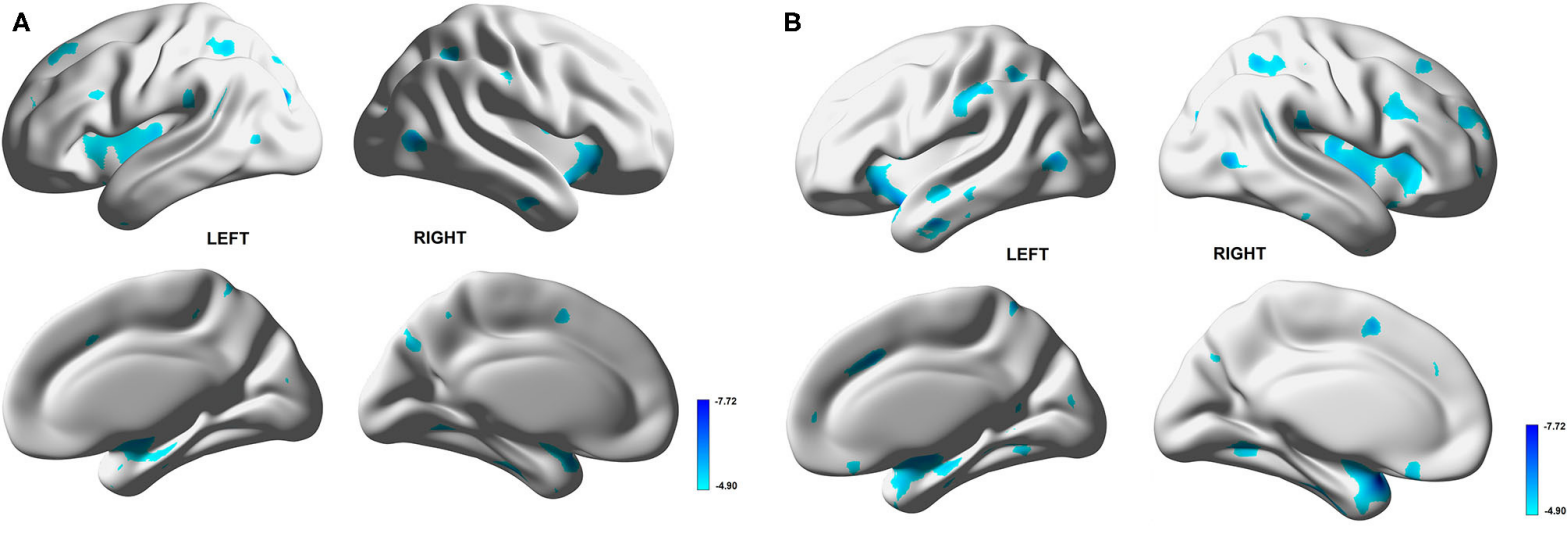
Voxel-based correlation between gray matter volume and mean vessel diameter (mVD) or vessel size index (VSI) values in white matter. Multiple gray matter regions, dominantly medial temporal lobes and insular cortices, show significantly negative correlation with mVD **(A)** and VSI **(B)**.

The results of the voxel-based correlation between mVD or VSI value and gray matter volume show that the multiple gray matter regions, including medial temporal lobes and the insular cortex, were significantly negatively correlated with the mVD and VSI (Figure 4). The correlation with non-adjusted age and TIV also presented that the multiple gray matter regions, including medial temporal lobes and insular cortex were significantly negatively correlated with the mVD and VSI (Supplementary Figure 3) Both mVD and VSI showed a significantly negative relation with TGV in correlation without adjustment of age and TVI (*r* = −0.529, and −0.647, *p* < 0.05, respectively). However, only VSI was negatively associated with the TGV in the correlation analysis with adjustment. The *r* partial values with TGV were −0.415 (*p* = 0.07) and −0.446 (*p* < 0.05), respectively.

## Discussion

In this clinical study, we performed *in vivo* vessel size imaging for deeper insight into the underpinnings of microvascular alterations in SVaD. The mVD and VSI were significantly higher in the white matter of SVaD compared to controls regardless of WMH lesions and could discriminate between SVaD and controls. Elevation of these parameters in SVaD patients were multifocally distributed in the supratentorial brain white matter and deep gray matter. Microvessel parameters associated with gray matter volume loss suggest that microvascular changes of the white matter eventually affect cortical function.

### SVD in Vascular Dementia

Because SVaD is a serious manifestation of SVD, we need to address the pathophysiology and advanced imaging modality of SVD to understand the results of this study. The extent of WMH in the brain is an important diagnostic indicator of the severity of SVD. In neuropathological studies, MRI shows that WMH is composed of gliosis, axonal loss, demyelination, and neuronal loss (17, 18). However, pathological studies are usually conducted in late-stage disease (19). In earlier stages, SVD begins with the dysfunction of endothelial cells associated with genetic factors, hypertension, or other systemic or environmental risk factors. Leakage across the blood–brain barrier (BBB) results in perivascular tissue and arteriolar wall damage (18, 20); consequently, arterioles lose their autoregulatory abilities to contract and expand, thereby regulating the blood supply to meet the brain's demand. Vessel stiffness increases pulsatility and diminishes the fluid flowing to the perivascular spaces (or interstitial fluid flushing) (5). This dysfunction also interferes with capillary flow homogenization, which disturbs the maintenance of efficient oxygen extraction (21). Oxidative stress promoted by hypoxia then leads to mitochondrial dysfunction, neuronal damage, and apoptosis through the nitric oxide synthase pathway (22). Therefore, most researchers believe that SVD-related brain damage begins with vascular dysfunction and interstitial edema (23). These findings, unfortunately, are not readily visible on conventional imaging.

In several studies, dynamic contrast-enhanced (DCE) perfusion MRI and diffusion tensor imaging (DTI) MRI show microstructural changes in the so-called NAWM, which worsened with SVD lesions. The parameters derived from DCE MRI, including Ktran, Vp, and signal enhancement maps (Et), represent leakage of contrast media into the interstitial space from the circulation through the compromised BBB integrity. These parameters allow clinicians to distinguish normal-appearing SVD burden from the healthy white matter (18). Free water imaging derived from DTI is an emerging technique that can accurately reveal extracellular water within the white matter. This feature is thought to be an excellent way to distinguish early stage SVD from controls (24). These techniques mainly focus on detecting changes in the BBB function as well as in nerve fibers and fluid content in the white matter.

In addition to BBB breakdown and subsequent vasogenic edema, morphological variations and dysfunction of cerebral microvessels are as important in the pathophysiologic axis of SVD-induced brain damage. Because microvascular changes precede parenchymal damage, from a therapeutic perspective, diagnosing early vascular damage in SVD is more valuable than recognizing gross changes of the white matter. However, there is no established method to directly visualize the vascular network on a microvascular scale. Therefore, we explored the vessel size imaging modality that reflects the properties of the local microvascular structures at the imaging scale for SVD (10).

### mVD and VSI Can Discriminate SVaD From Control

Of four microvessel parameters driven from vessel size imaging, only mVD and VSI were significant in the statistical analyses in this study. Because mVD increases as the vessel size increases, it can be used as a measure of the average vessel size within an ROI or a voxel (25). VSI is the mean vessel radius averaged over the capillary population with the weight of its volumetric fraction (10). Although it correlates with mVD, it also accounts for the water diffusion rate and is more quantitative compared to mVD. Therefore, it can help monitor microvessel dilation and contraction. Our results showed that mVD and VSI were sensitive parameters that could detect differences between the two groups. There are two possible explanations for these results. One is that smaller arterioles occlude or collapse from neurovascular unit dysfunction or arteriolosclerosis although larger arterioles can preserve their function and caliber in SVD (22). The other hypothesis is that impaired oxygen supplies (due to BBB dysfunction) cause vasodilatation through autoregulation of the neurovascular network. These hypotheses were explained in previous autopsy studies of older patients with dementia. In a postmortem study, the capillaries in the white matter of patients with dementia were significantly wider than those in the white matter of normal patients (26). Another human pathological study showed that the death of vascular endothelial cells associated with blood flow shear stress leads to capillary collapse and formation of bundle capillaries and tortuous vessels of a larger caliber (27).

We simultaneously investigated vessel size imaging in an the AD model mouse brain using 7.0T MRI. A major causal mechanism of AD was alteration in the BBB and dysfunction of neurovascular autoregulation. We found that VSI was significantly higher in the AD model mouse group than in the control mouse group in the somatosensory cortex area. This finding suggests that imaging findings in the AD mouse model might be related to the vascular pathology or damage to the neurovascular unit, consistent with our human study. Our research suggests that mVD and VSI can be used as markers of neurovascular dysfunction and may also be useful in the early diagnosis of microvascular compromise in neurodegenerative diseases.

### Relation Between Microvessel Parameters and Gray Matter Volume

The volume of WMH in patients with small vessel disease is related to the gray matter volume loss and cognition function (28, 29). The inverse relationship between microvessel parameters in the white matter and the gray matter volume loss in the current study supports the initial pathophysiological hypothesis that the former pathology precedes the latter. Most researchers believe that SVD-related brain damage begins with vascular dysfunction and interstitial edema (23). These findings, unfortunately, are not readily visible on conventional imaging. The current study suggests that mVD or VSI can be useful to predict the brain damage and cognitive function in SVD. Therefore, further studies are necessary to evaluate how microvessel parameters can affect the gray matter and cognitive function in early stages of SVD or subclinical stages of SVaD.

### Limitations and Further Extensions

Our study has several limitations. First, the sample size was small and only included patients with SVaD. Second, this preliminary prospective study was performed using limited imaging conditions and specific contrast media because it did not account for comorbid variables that could affect vascular diseases, such as hypertension, diabetes, and sex. In this study, we used a Gadolinium-based contrast agent. Most previous animal studies were performed with an intra-vascular pool contrast agent, such as superparamagnetic iron oxide nanoparticles. Third, we only included patients with established SVaD. Therefore, it is unclear if our findings can be applied as early diagnostic criteria to identify SVaD. Fourth, structural changes could not be distinguished from functional changes in microvessels (e.g., VSI or mVD) in this study. Fifth, ROI analyses have the risk of bias that ROI drawn in DWM might include the different patterns of the microvascular network, such as the cortex and juxtacortical white matter.

Our study also has several strengths. For instance, to our knowledge, this is the first *in vivo* human imaging study of neurovascular changes reflecting neurodegeneration with cortical thinning and cognitive decline that can be applied in clinical practice. We find that VSI can potentially be used in the early diagnosis of SVD and in detecting tumors and ischemia. Nevertheless, further larger-scale studies are required to investigate SVD and provide more precise information regarding its clinical utility in this setting. Future studies are also required to determine the correlation between VSI and risk factors of SVD, such as hypertension. Finally, future studies must validate the early diagnosis of SVD and confirm its efficacy in the long term.

### Further Refinement and Implementation

The microvascular mapping techniques implemented in this study depend on the qualities of quantifications of mapping R2 and R2^*^ relaxation rates and the ADC value. First, to obtain reliable R2 and R2^*^ maps, we need to acquire a lot of echoes, which are reasonable to ~10 echoes or more. However, acquisitions of more echoes can prolong the scan time, and some patients may be not able to scan a long time. Therefore, the rationale behind choosing the number of echoes to scan patients should be further refined. Second, Δ*R*_2_ and $\Delta R_{2^{*}}$ are the differences of R2 and R2^*^ relaxation rates, respectively, before and after the injection of a contrast agent. R2^*^ maps are sensitive to the contrast agent; therefore, a reliable Δ*R*2^*^ value can be obtained. However, R2 maps are relatively less sensitive than R2^*^ maps with a contrast agent. Therefore, we should be careful to fit images to obtain an R2 map. Third, VSI is also dependent on the ADC value. In most clinical studies, DWI with *b*-values of 0 and 1,000 are acquired. However, to investigate a microvascular signal, we may acquire a DWI signal with relatively high and several *b-*values to fit a reliable ADC map. This issue should be refined in future studies. Finally, in this study, we used a Gadolinium-based contrast agent rather than superparamagnetic iron oxide (SPION) nanoparticles because it is allowed to be implemented in clinical studies. However, to obtain a reliable microvascular structure, an intravascular pool contrast agent such as SPION is required. Therefore, in future studies, comparisons of microvascular structure between the two different contrast agents should be evaluated in patients.

## Conclusions

We investigate the cerebral microvascular changes in SVaD using *in vivo* MRI techniques and find that mVD and VSI are sensitive to differences between SVaD and controls and that vessel size imaging may serve as an effective biomarker of neurodegenerative diseases. Interestingly, these parameters show a significant negative correlation with the amount of gray matter. These results suggest that the microvascular damage in SVD may precede gray matter damage. Future studies are required to validate the use of these early diagnostic tools for SVD and to consider other potential variables.

## Data Availability Statement

All datasets generated for this study are included in the article/Supplementary Material.

## Ethics Statement

The studies involving human participants were reviewed and approved by Institutional Review Board, kyung Hee University Hospital at Gangdong. The patients/participants provided their written informed consent to participate in this study.

## Author Contributions

C-WR conceived of the presented idea and supervised the project. G-HJ developed the theory and verified the analytical methods. H-IC and SK carried out the measurement and experiment. H-IC and HR wrote the manuscript with support from C-WR and G-HJ. All authors discussed the results and contributed to the final manuscript.

## Conflict of Interest

The authors declare that the research was conducted in the absence of any commercial or financial relationships that could be construed as a potential conflict of interest.

## Abbreviations

SVaD: subcortical vascular dementia
mVD: mean vessel diameter
VSI: vessel size index
GMV: gray matter volume
TIV: total intracranial volume
FDR: false discovery rate
WMH: white matter hyperintensity
NAWM: normal appearing white matter
IQR: interquartile range.
