# Supplementary material for: Changes in Microvascular Morphology in Subcortical Vascular Dementia: A Study of Vessel Size Magnetic Resonance Imaging
Source: Front Neurol. 2020 Oct 29;11:545450. doi: 10.3389/fneur.2020.545450 (PMC7658467; doi:10.3389/fneur.2020.545450)

Supplementary Figure 1. Four microvessel index maps calculated based on pre- and post-TSE and GE images and ADC map

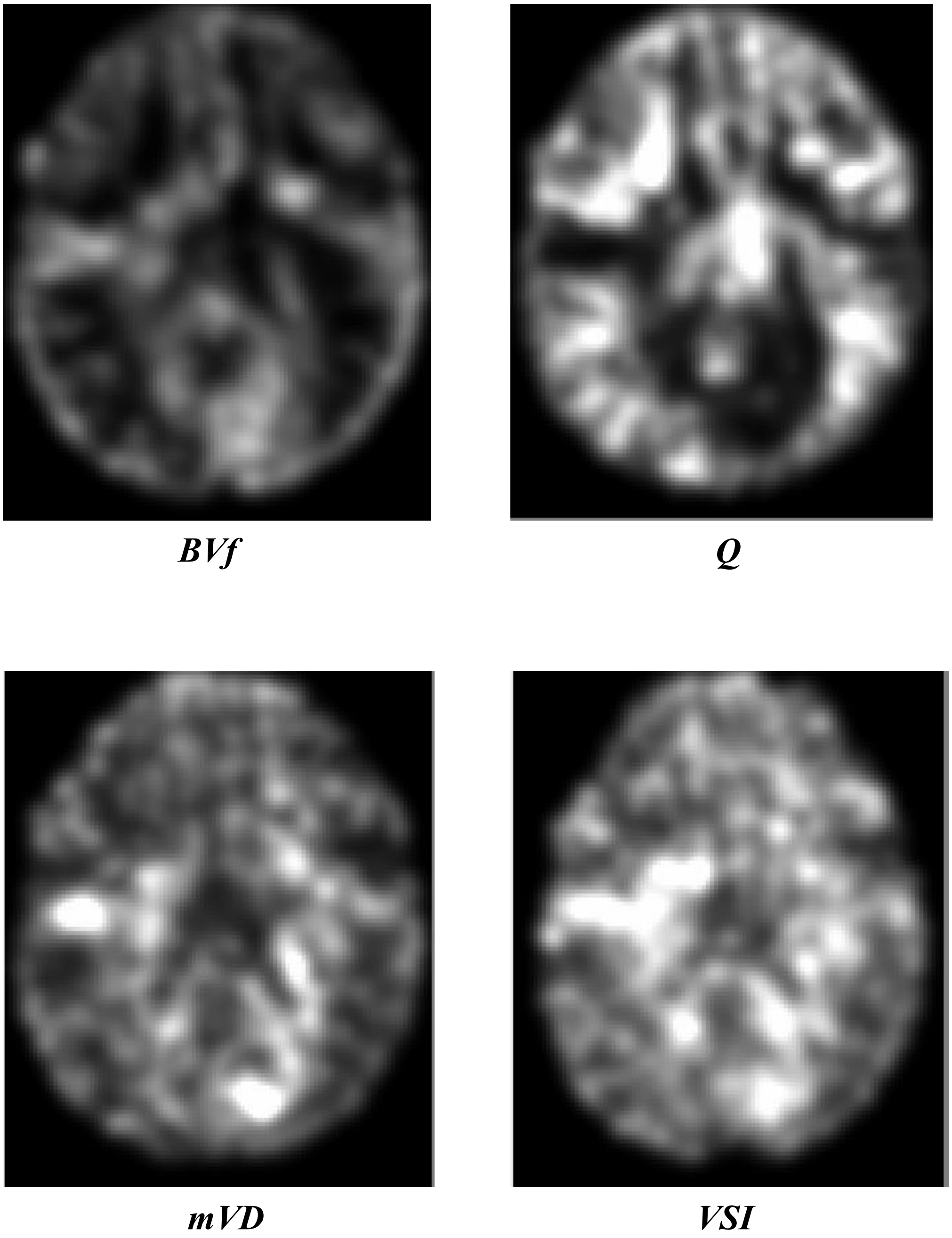

Supplement: Supplementary file 1 [file Image_1.pdf]
