# Supplementary material for: Changes in Microvascular Morphology in Subcortical Vascular Dementia: A Study of Vessel Size Magnetic Resonance Imaging
Source: Front Neurol. 2020 Oct 29;11:545450. doi: 10.3389/fneur.2020.545450 (PMC7658467; doi:10.3389/fneur.2020.545450)
Supplement: Supplementary file 2 [file Image_2.pdf]

Supplementary Figure 2. Receiver-operating characteristic (ROC) curve is constructed to determine the mVD and VSI of discriminating between patients with SVaD and controls

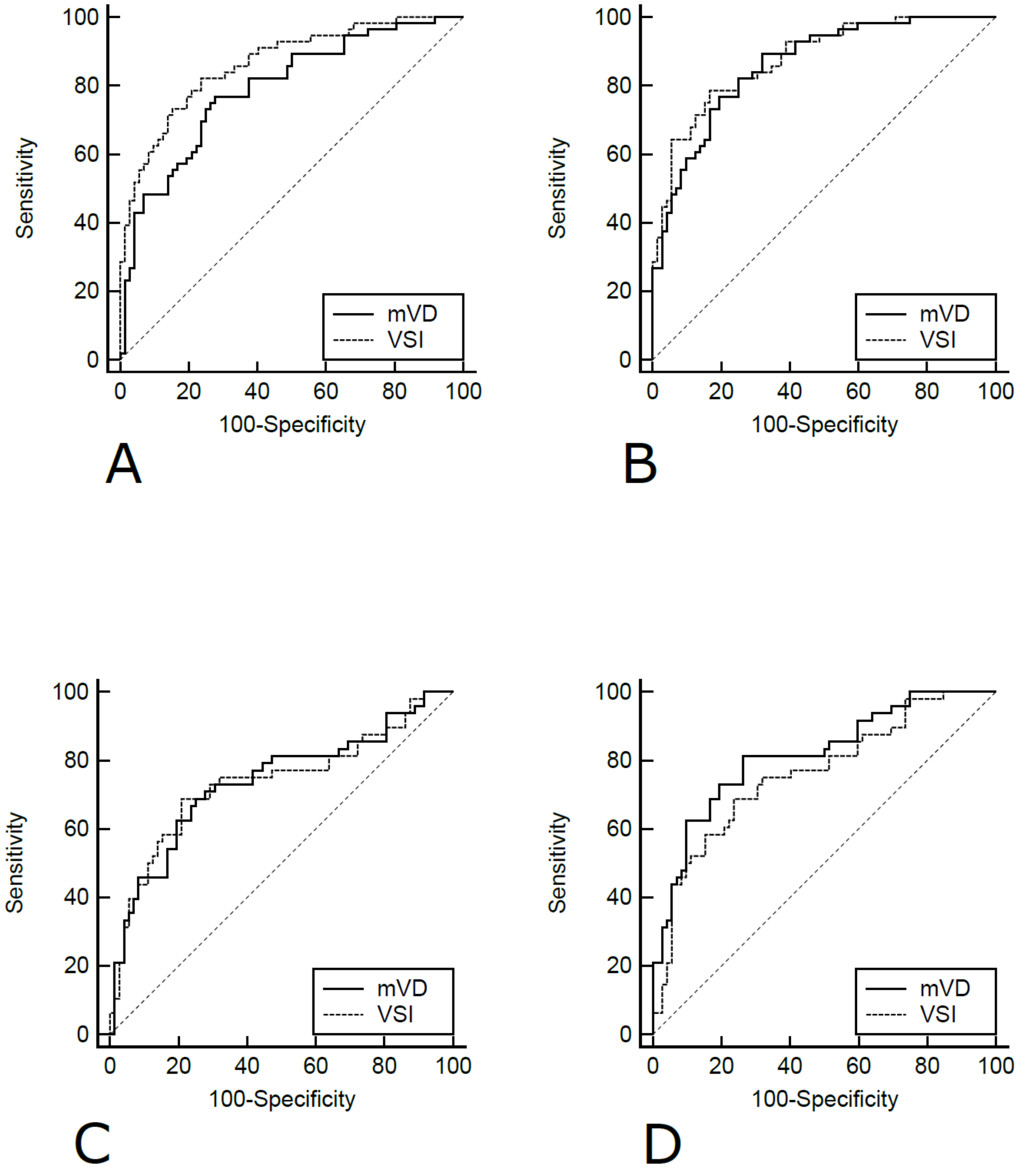

A. mVD and VSI of discriminating between ROIs in whiter matter hyperintensity (WMH) of SVaD and ROI in periventricular white matter (PVWM) of controls.

B. mVD and VSI of discriminating between ROIs in WMH of SVaD and ROI in deep white matter (DWM) of controls.

C. mVD and VSI of discriminating between ROIs in normal appearing white matter (NAWM) of SVaD and ROI in PVWM of controls.

D. mVD and VSI of discriminating between ROIs in NAWM of SVaD and ROI in DWM of controls.
