# Supplementary material for: Changes in Microvascular Morphology in Subcortical Vascular Dementia: A Study of Vessel Size Magnetic Resonance Imaging
Source: Front Neurol. 2020 Oct 29;11:545450. doi: 10.3389/fneur.2020.545450 (PMC7658467; doi:10.3389/fneur.2020.545450)

**Supplementary Figure 3.** Voxel-based correlation (without adjusted age and TIV)between gray matter volume and mean vessel diameter (mVD) or vessel size index (VSI) values in white matter. Multiple gray matter regions, dominantly medial temporal lobes and insular cortices, shows significantly negative correlation with mVD (A) and VSI (B).

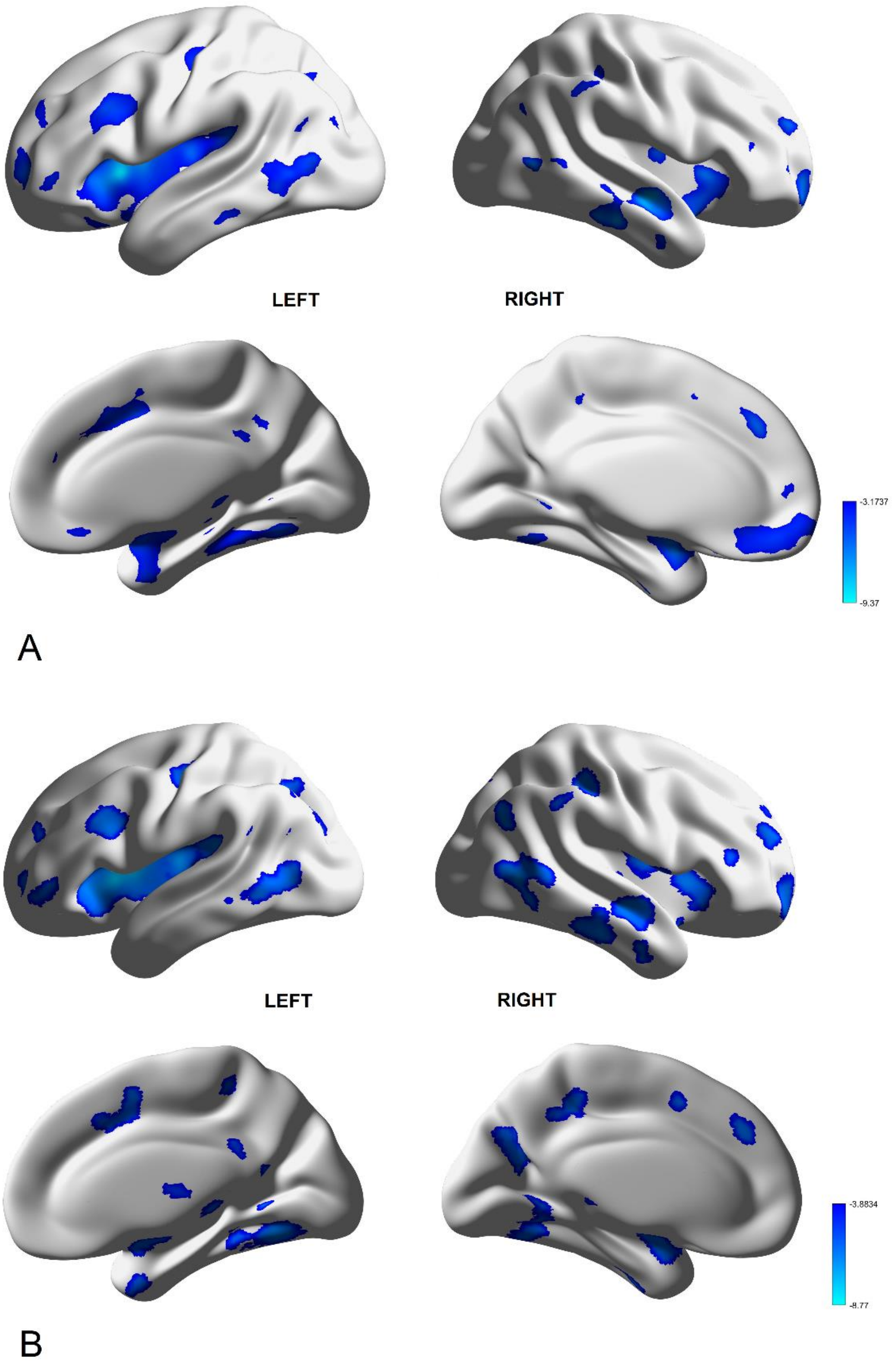

Supplement: Supplementary file 3 [file Image_3.pdf]
